# Environmental convergence in facial preferences: a cross-group comparison of Asian Vietnamese, Czech Vietnamese, and Czechs

**DOI:** 10.1038/s41598-020-79623-1

**Published:** 2021-01-12

**Authors:** Ondřej Pavlovič, Vojtěch Fiala, Karel Kleisner

**Affiliations:** Department of Philosophy and History of Science, Faculty of Science, Charles University, Vinicna 7, Prague, 128 44 Czech Republic

**Keywords:** Behavioural ecology, Psychology and behaviour

## Abstract

It has been demonstrated that sociocultural environment has a significant impact on human behavior. This contribution focuses on differences in the perception of attractiveness of European (Czech) faces as rated by Czechs of European origin, Vietnamese persons living in the Czech Republic and Vietnamese who permanently reside in Vietnam. We investigated whether attractiveness judgments and preferences for facial sex-typicality and averageness in Vietnamese who grew up and live in the Czech Republic are closer to the judgements and preferences of Czech Europeans or to those of Vietnamese born and residing in Vietnam. We examined the relative contribution of sexual shape dimorphism and averageness to the perception of facial attractiveness across all three groups of raters. Czech Europeans, Czech Vietnamese, and Asian Vietnamese raters of both sexes rated facial portraits of 100 Czech European participants (50 women and 50 men, standardized, non-manipulated) for attractiveness. Taking Czech European ratings as a standard for Czech facial attractiveness, we showed that Czech Vietnamese assessments of attractiveness were closer to this standard than assessments by the Asian Vietnamese. Among all groups of raters, facial averageness positively correlated with perceived attractiveness, which is consistent with the "average is attractive" hypothesis. A marginal impact of sexual shape dimorphism on attractiveness rating was found only in Czech European male raters: neither Czech Vietnamese nor Asian Vietnamese raters of either sex utilized traits associated with sexual shape dimorphism as a cue of attractiveness. We thus conclude that Vietnamese people permanently living in the Czech Republic converge with Czechs of Czech origin in perceptions of facial attractiveness and that this population adopted some but not all Czech standards of beauty.

## Introduction

Throughout the history of humankind, various peoples migrated to parts of the world where they were visually distinct from the standards of facial appearance of local majority population. This was also the case of Vietnamese citizens who were coming to Czechoslovakia since 1956 to be trained in mechanical engineering, light industries, or to study at Czechoslovak universities^1^. The arrival of Vietnamese students, trainees, and guest workers was based on friendly diplomatic and economic contacts between the Czechoslovak Socialist Republic and the Democratic Republic of Vietnam. Many of these immigrants did not learn the Czech language, which somewhat isolates this older wave of immigrants who arrived during the era of state socialism from the majority Czech population. After the fall of the Communistic regime in Czechoslovakia in 1989, many more Vietnamese came from Vietnam and from other European countries. After the Velvet Revolution, persons of Vietnamese origin found their niche within the Czech society as small traders in street markets or operators of small grocery shops. The young generation, that is, usually the offspring of the Vietnamese immigrants, is better integrated into Czech society and Czech educational system and many are promising students who successfully aspire to professional careers. They call themselves "the Banana generation"—meaning yellow outside, white inside—because unlike their parents and despite their Asian appearance, they speak fluent Czech. They sometimes feel more Czech than Vietnamese: they were born in the Czech Republic or lived there for most of their life, and they often have an ambivalent relationship to the country their parents came from. It is estimated that the Vietnamese minority in the Czech Republic currently numbers around 60,000. In this study, we recruited members of this younger generation of the Vietnamese minority as "Czech Vietnamese" raters.

One can learn a lot just by looking at another person's face. Apart from the most obvious information, such as sex and age, faces also provide information about strength, health, health risks, fighting performance, social status, emotional states, and various personality traits^2–7^. Facial perception is thus one of the most important aspects of human social interactions, especially due to its decisive role in forming first impressions^8–10^.

It is well documented that people are better at recognizing and remembering faces and reading emotional expressions in faces of own race than in faces belonging to other races^11–16^. This phenomenon is known as the *other-race effect*. It implies that the perception of various attributes of faces belonging to other-race faces is usually less effective than the recognition of attributes of own-race faces. In short, it is more difficult to distinguish and decode particular facial features and their meaning in faces of other ethnicities. These facial perception biases evolve in very early childhood^17–19^. It has been shown that own-race faces are perceived in a more holistic manner that other-race faces^20^. Face perception strongly depends on the viewing context and previous exposure^21^. Greater amount of individuating experiences with other-race faces tend to reduce the other-race effect in holistic processing^22^. On the other hand, recent research had shown that some social traits may be better assessed for outgroup faces^23^. It has been demonstrated that racially typical individuals are more vulnerable to various forms of social discrimination and stigmatization, especially by outgroup members^24^. Other-race (versus own-race) perception may be mediated by nonracial categorization. The people classified as in-group (versus out-group) are perceived in a more positive manner^25^. Own-race bias can be minimized or eliminated by positive emotions, which help promote a broader social identity prior to interpersonal interaction^26^. It has been demonstrated that social categorization on its own can elicit biases in facial recognition and perception and might be thus partially responsible for the other-race effect^27^. It seems that both sufficient perceptual experience with other-race faces and motivation to individuate out-group faces successfully reduces the other-race effect in perception^28^.

Attractiveness is among the most frequently studied attributes of face perception, because it plays a key role in both mate choice and social perceptions. Positive personality traits are cross-culturally listed among the most significant factors in mate choice by both sexes and attractive people tend to be attributed more positive qualities by others^29^. For example, attractive individuals are treated more positively than less attractive persons and are perceived as more healthy, intelligent, competent, and more experienced in dating (for a meta-analytic review of stereotypes associated with beauty, see^30^. There is a cross-cultural consensus on attractiveness standards of human faces^31,32^ although in various cultures, there may be a different degree of emphasis on various attractiveness features such variation in skin texture, skin color, eye and hair color, facial masculinity, or facial shape^33–40^. Among the traits which contribute to the resulting facial attractiveness the most are averageness (proximity to a population norm), facial symmetry, and sexual dimorphism (the degree of development of sexually dimorphic features).

More average (that is, less distinct) faces are perceived as more attractive due to stabilizing selection^41,42^. Facial averageness reflects higher genetic diversity, greater heterozygosity, and therefore also higher biological quality^29,43,44^. Male and female faces manipulated to greater averageness are regarded as healthier-looking^45^. Faces perceived as the most attractive seem to be those which are close to the average but not the most average ones^42,46–48^.

An alternative explanation of preference for typical faces might be that faces were regarded as attractive not in virtue of their proximity to the population norm but rather due to a symmetry resulting from computational averaging that takes place during the production of facial composites^49,50^. That explanation, however, fails to account for the association between attractiveness and averageness calculated as the distance of natural faces from their population mean, which is a method used in this study. When averageness was computed as a Procrustes distance from the average face, higher averageness was positively linked with attractiveness^51^. Moreover, in studies which used composites, averageness accounted for a significant part of attractiveness even after exclusion of the effect of symmetry^52^.

In human faces, sexual dimorphism develops around puberty. This process is driven by increasing influence of sex steroids (such as estrogens, androgens, and progestogens, usually listed only as testosterone and estrogens). It has been reported that feminine facial shapes in females are perceived as more attractive both between and within cultures^36,43,53–55^. In virtue of its association with high estrogen levels, facial femininity in women may indicate sexual maturity and high reproductive capacity^43,56^ or, due to its association with youthfulness, it may serve as a cue to residual reproductive capacity^57^. With respect to links between attractiveness and individual expression of sexual dimorphism in male faces, on the other hand, reported results are inconsistent. Some studies reported women's preference for more masculine faces^58,59^ while other studies found evidence of preference for more feminine male faces^60–62^. It seems that both masculine and feminine male faces might be preferred, whereby the actual preference depends on an interaction between perceived hormone markers and the hormonal state of the female perceiver^63,64^. Moreover, it has been shown that preference for sexually dimorphic traits varies among human cultures and its association with attractiveness may have emerged relatively recently in human evolution, in particular with the appearance of urban Western societies^65^. Alternatively, the variation over preference for sexual dimorphism among different populations can also be explained by other factors such as differences in national health, income inequality, and homicide rate^66,67^.

Age serves as a cue to actual and residual fertility in women^68^ and, to a lesser extent, also in men^69^. In women, the decline of attractiveness with age is steeper and seems stable across cultures^70–72^. Even in young women, the current attractiveness is negatively associated with their estimated age at menopause (based on woman's mother menopause age)^73^. In men, the attractiveness declines with age more slowly. Middle-aged men may be attractive, as they possess higher status and more resources while keeping a sufficient level of fertility^71^.

The actual relative weight (body mass index) predicted a person's perceived adiposity and perceived weight^74,75^, though the viewing angle may bias the perception of weight from the face^76^. Both high and low perceived facial adiposity (underweight and overweight) lowers perceived healthiness. High perceived facial adiposity is also associated with worse actual health^74^. Higher BMI is negatively associated with perceived female bodily^77^ and facial attractiveness^78^. Nonetheless, there are cross-cultural differences in the most preferred relative weight^79^. In this study, we investigate differences in perceived attractiveness of European faces of Czech origin between Vietnamese persons living in the Czech Republic (henceforth Czech Vietnamese) and Vietnamese permanently residing in Vietnam (henceforth Asian Vietnamese). We expect that attractiveness judgments of Czech Vietnamese raters should be closer to attractiveness judgments of Czech European raters than to assessments by Asian Vietnamese raters. In other words, we hypothesize that the correlation in attractiveness judgments between Czech European and Czech Vietnamese raters is stronger than the correlation between Czech European and Asian Vietnamese raters. Moreover, we expect that this difference should be statistically significant. We also expect that Czech Vietnamese raters should be better adapted to the perception of sexually dimorphic traits of Czech faces and to local morphological typicality in general than Asian Vietnamese raters. In other words, we expect that the Czech Vietnamese raters have a better representation of both Czech facial population mean and the degree of facial masculinization/feminization and that the correlation between attractiveness and degree of shape sexual dimorphism (SShD) measured from faces will be greater between Czech and Czech Vietnamese than between Czech and Asian Vietnamese raters. Finally, we investigate the pattern of relative contribution of objectively measured level of sexual dimorphism and averageness to the perception of facial attractiveness in all three groups of raters (Czech Europeans, Czech Vietnamese, and Asian Vietnamese).

## Materials and methods

### Ethical statement

All the experiment protocol for involving humans was in accordance to guidelines of national/international/institutional or Declaration of Helsinki. All procedures mentioned and followed were approved by the Institutional Review Board of the Faculty of Science of the Charles University (protocol ref. number 06/2017).

### Data acquisition

We took facial portraits of 100 Czech participants (50 women: mean age = 23.64; SD = 4.29; range = 19–36; 50 men: mean age = 24.04; SD = 3.88; range = 19–34). Portraits were collected in Prague, Czech Republic, during several sessions but always in the same room under the same conditions.

Facial portraits were acquired using a standardized procedure^80^. Participants were photographed in front of a white background with a full-frame color camera Canon 6D using a studio flash. We used an EX SIGMA 1.4f 85 mm lens and set the focus point to the left eye. Shutter speed was set to 1/100 s, exposure to ISO 100, aperture F8, and 2/3 of strobe power. Photographs were taken from a tripod adjusted to each participant's height so that the target's face was in the middle of the image. Distance between lens and target's tip of the nose was set to 1.25 m to preserve natural variability in face size in each image and to obtain the sharpest result possible with the lens used. Participants were instructed to adopt a neutral, non-smiling facial expression and refrain from any mimic muscle activity. Participants were asked not to wear any makeup, glasses, jewelry, or other decorations and they all wore a black T-shirt.

### Rating of facial images

Facial portraits were assessed for attractiveness by Czech European raters, members of the Vietnamese minority living in the Czech Republic and Vietnamese persons living in Vietnam. In Vietnam, raters were students of the University of Science and Technology of Hanoi. In Czech Republic, raters were Czech university students of European origin. The Czech Vietnamese raters were attendees of the special meeting called "Banana Fest" a multi-genre festival introducing the culture of so-called "banana children", which is a colloquial name for the second generation of Vietnamese migrants living in the Czech Republic. The inclusion criteria for Czech Vietnamese were the participant's ability to fluently speak Czech and the information that they attended Czech education system. The participants from all three rater-groups were asked to report their sexual orientation (heterosexual, homosexual, other). We used only data from individuals that identify themselves as heterosexuals.

Photographs of Czech European women were rated by Czech European men (N = 34, mean age = 28.18, SD = 4.210), Czech Vietnamese men (N = 28, mean age = 23.04, SD = 6.19) and Asian Vietnamese men (N = 53, mean age = 21.18, SD = 2.01). Photographs of Czech men were rated by Czech European women (N = 89, mean age = 27.56, SD = 4.23), Czech Vietnamese women (N = 29, mean age = 24.17, SD = 7.44) and Asian Vietnamese women (N = 32, mean age = 21.9, SD = 4.33). In other words, raters of each sex rated a set of 50 facial portraits of the other sex for attractiveness: men rated photographs of women and vice versa. Raters viewed each portrait on an IPS computer screen with resolution 1980 × 1080 pixels, using a fullscreen setup of survey session, seeing only one photograph at the time, and assessed attractiveness on a 7-point scale (ranging from 1—very unattractive to 7—very attractive). There was no time limit for exposure to each portrait. In each rating session, the order of photographs was randomized.

Cronbach’s alpha showed high interrater agreement for attractiveness judgments of Czech female photographs by male Czech European raters (N = 33, α = 0.961), male Czech Vietnamese raters (N = 27, α = 0.982), and male Asian Vietnamese raters (N = 57, α = 0.984). Female raters also showed high interrater agreement when judging the attractiveness of Czech male photographs (female Czech European raters: N = 89, α = 0.971; female Czech Vietnamese raters: N = 16, α = 0.972; female Asian Vietnamese raters: N = 32, α = 0.962).

All participants provided their informed consent by clicking on the 'I agree' button to consent with their participation in the study.

### Statistical analyses

Relationships between variables were preliminarily explored using Pearson's zero-order correlation coefficient. Average ratings within each rater group (Czech European, Czech Vietnamese, and Asian Vietnamese) were first calculated for individual facial images.

To analyze the data on the level of individual ratings, we built a mixed effect model using the *lmer* function within the *lmerTest* R package^81^, with completely specified varying intercepts and slopes among rater groups and random intercepts of raters within rater groups and random intercepts of rated targets (faces). Attractiveness ratings were set as a response variable and age, BMI, averageness, and sexual shape dimorphism as predictors. Varying intercepts and slopes were included as interaction terms between predictors and rater groups. We evaluated contrasts between pairs of rater-groups using *glht* function from *multcomp* R package^82^. Two separate models were built, one for men and one for women.

In addition to mixed effect models, we used path analysis to investigate directed dependencies between perceived attractiveness, sexual shape dimorphism (SShD), averageness, individual's age, and body mass index (BMI). Path analyses were conducted using the *sem* function within the *lavaan* package available in R software for statistical computing^83^. First, we predicted directed dependencies among the variables (no correlational associations were investigated). Dependencies were directed according to logical constraints, so that for instance age could either positively or negatively affect the BMI but BMI could not affect a person's chronological age. No latent variables were included in the model syntax.

The main dependent variable was attractiveness. SShD, BMI, and averageness were set as mediators between age and attractiveness. Averageness and sexual dimorphism were also viewed as mediators in the association between BMI and attractiveness. A direct association between age and attractiveness was also included in the model. The number of observations was relatively low, which is why we calculated a "robust" p-value using the Monte Carlo permutation procedure. We estimated a full model fit on randomized datasets based on 10,000 simulation runs, on which we derived the distribution of expected regression coefficients.

### Geometric morphometrics and anthropometric measurements

A total of 72 landmarks were digitized on the faces of 50 men and 50 women. Not all aspects of facial morphology can be described by discrete homologous points that can be unambiguously identified in every face in the sample. For this reason, 36 landmarks were identified a posteriori as semilandmarks that denote curves and outlines. Facial configurations were aligned by generalized Procrustes analysis using the *gpagen* function in R geomorph package^84^. Procrustes-aligned specimens were projected onto tangent space prior to further computations. An algorithm which reduces the bending energy between each facial configuration and Procrustes mean was used to optimize the semilandmarks positions.

To measure the level of individual expression of SShD, we applied the Procrustes fit (a generalized Procrustes analysis) to all 100 faces (both men and women). Subsequently, we determined the position of each individual facial shape along the axis connecting male and female facial averages^85,86^. By projecting each individual face onto a vector linking the average female and male face, we obtained SShD scores which were used in subsequent analyses.

To calculate facial averageness, we performed a Procrustes fit separately for male and female faces. Average landmark configuration (consensus) was then calculated separately for male and female faces. Procrustes distances between the consensus and each facial configuration in the set were computed and used as a measure of individual averageness. Lower facial averageness scores thus indicate particular configuration's proximity to the mean shape, while higher averageness scores indicate more distinct faces.

The weight of the photographed person was taken by scales, and the height measured by tape measure fixed on the wall. We calculated the body mass index (BMI) of a given person as weight [kg] divided by squared height [m]. The age of the person was self-reported.

## Results

Pearson's correlations between attractiveness ratings and anthropometric traits are summarized in Supplementary Tables S1 and S2 for photos of men and women, respectively. The results were adjusted for multiple tests.

The results of mixed effects models are summarized in Tables 1 and 2. The attractiveness ratings of Czech Europeans were used as a standard measure of Czech facial attractiveness. Attractiveness ratings of Czech European and Czech Vietnamese raters were associated more strongly that the ratings of Czech Vietnamese and Asian Vietnamese raters.

**Table 1:** Summary of the results of mixed effects modelling for faces of men (female raters).

Random effects							
Groups	Name	Variance	SD	Corr			
Ratergroup:rater	(Intercept)	0.523	0.723				
	age	0.002	0.045	− 0.67			
	bmi	0.001	0.028	− 0.35	0.93		
	avrg	0.001	0.029	− 1.00	0.66	0.34	
	sshd	0.001	0.034	− 0.61	− 0.18	− 0.53	0.62
Face	(Intercept)	0.558	0.747				
Residual		0.798	0.893				

Fixed effects	Estimate	SE	df	t-value	p-value		
(Intercept)	2.779	0.131	96.553	21.186	< 0.001***		
age	− 0.029	0.114	45.752	− 0.253	0.802		
bmi	− 0.058	0.114	45.659	− 0.506	0.615		
avrg	− 0.222	0.109	45.668	− 2.033	0.048*		
sshd	− 0.081	0.109	45.697	− 0.745	0.460		
ratergroupCZVN	− 0.258	0.157	147.004	− 1.644	0.102		
ratergroupAVN	0.406	0.151	147.012	2.685	0.008**		
age:ratergroupCZVN	− 0.070	0.031	345.605	− 2.296	0.022*		
age:ratergroupAVN	− 0.036	0.029	345.846	− 1.205	0.229		
bmi:ratergroupCZVN	− 0.014	0.029	802.030	− 0.462	0.644		
bmi:ratergroupAVN	− 0.063	0.028	802.854	− 2.212	0.027*		
avrg:ratergroupCZVN	0.002	0.028	1275.341	0.086	0.932		
avrg:ratergroupAVN	0.056	0.027	1278.423	2.064	0.039*		
sshd:ratergroupCZVN	− 0.027	0.028	392.408	− 0.943	0.346		
sshd:ratergroupAVN	− 0.049	0.027	392.979	− 1.790	0.074		

Significance levels: *p < 0.05 **p < 0.01 ***p < 0.001.

*CZVN* Czech Vietnamese, *AVN* Asian Vietnamese, *sshd* sexual shape dimorphism, *avrg* distance from the average, *bmi* body mass index.

**Table 2:** Summary of the results of mixed effects modelling for faces of women (male raters).

Random effects							
Groups	Name	Variance	SD	Corr			
Ratergroup:rater	(Intercept)	0.520	0.721				
	age	0.005	0.073	− 0.23			
	bmi	0.001	0.038	− 0.20	1.00		
	avrg	0.012	0.109	0.07	0.96	0.96	
	sshd	0.000	0.020	0.04	0.96	0.97	1.00
Face	(Intercept)	0.258	0.508				
Residual		0.791	0.889				

Fixed effects	Estimate	SE	df	t-value	p-value		
(Intercept)	2.681	0.146	152.60	18.323	< 0.001***		
age	− 0.201	0.077	53.65	− 2.626	0.011*		
bmi	− 0.210	0.076	51.71	− 2.751	0.008**		
avrg	− 0.436	0.078	57.12	-5.584	0.000***		
sshd	− 0.092	0.076	51.19	− 1.206	0.233		
ratergroupCZVN	− 0.115	0.188	111.00	− 0.610	0.543		
ratergroupAVN	0.487	0.162	111.00	2.997	0.003**		
age:ratergroupCZVN	− 0.012	0.038	158.30	− 0.326	0.745		
age:ratergroupAVN	0.032	0.032	158.40	1.002	0.318		
bmi:ratergroupCZVN	0.042	0.034	399.90	1.224	0.222		
bmi:ratergroupAVN	0.103	0.030	400.20	3.479	0.001***		
avrg:ratergroupCZVN	0.129	0.043	124.20	2.996	0.003**		
avrg:ratergroupAVN	0.174	0.037	124.30	4.695	< 0.001***		
sshd:ratergroupCZVN	26.810	0.015	1526.00	0.274	0.784		
sshd:ratergroupAVN	− 0.020	0.008	536.50	0.991	0.322		

Significance levels: *p < 0.05, **p < 0.01, ***p < 0.001.

*CZVN* Czech Vietnamese, *AVN* Asian Vietnamese, *sshd* sexual shape dimorphism, *avrg* distance from the average, *bmi* body mass index.

Post hoc comparisons of selected pairs of rater-groups (using the Tukey HSD test) indicated that the attractiveness assessment of Asian Vietnamese women was significantly different from Czech Europeans (δ _CZ−AVN_ = − 0.406, SE = 0.151, p = 0.02) as well as from Czech Vietnamese female raters (δ _CZVN−AVN_ = − 0.6641, SE = 0.188, p = 0.001). Czech European and Czech Vietnamese female raters did not significantly differ in their attractiveness judgment of Czech European male faces (δ _CZ−CZVN_ = − 0.258, SE = 0.157, p = 0.224).

In similar vein, the attractiveness assessment of Asian Vietnamese was significantly different from Czech Europeans (δ _CZ−AVN_ = − 0.487, SE = 0.1624, p = 0.008) as well as from Czech Vietnamese male raters (δ _CZVN−AVN_ = − 0.602, SE = 0.171, p = 0.001). Czech European and Czech Vietnamese male raters did not significantly differ in their attractiveness perception of Czech European female faces (δ _CZ–CZVN_ = − 0.115, SE = 0.188, p = 0.814).

The preference for average male faces was statistically different neither between Czech European and Czech Vietnamese (δ _CZ–CZVN_ = 0.002, SE = 0.028, p = 0.995) nor between Czech European and Asian Vietnamese (δ _CZ–AVN_ = 0.056, SE = 0.027, p = 0.096,) as well as between Czech Vietnamese and Asian Vietnamese (δ _CZVN–AVN_ = 0.054, SE = 0.034, p = 0.248) female raters. In case of female faces, the preference for faces with higher averageness was significantly different between Czech European and Czech Vietnamese (δ _CZ–CZVN_ = − 0.13, SE = 0.043, p = 0.008) and between Czech European and Asian Vietnamese (δ _CZ−AVN_ = − 0.174, SE = 0.037, p < 0.001) male raters. Averageness preference did not differ significantly between Czech Vietnamese and Asian Vietnamese (δ _CZVN–AVN_ = − 0.05, SE = 0.039, p = 0.475) male raters.

The preference for facial dimorphism did significantly differ between in neither of compared rater groups for both male (δ _CZ–CZVN_ = 0.027, SE = 0.028, p = 0.61; δ _CZ–AVN_ = − 0.049, SE = 0.027, p = 0.17; δ _CZVN–AVN_ = − 0.022, SE = 0.034, p = 0.788) and female photos (δ _CZ–CZVN_ = 0.009, SE = 0.033, p = 0.959; δ _CZ–AVN_ = 0.028, SE = 0.028, p = 0.581; δ _CZVN–AVN_ = 0.019, SE = 0.03, p = 0.797).

**Figure 1:**
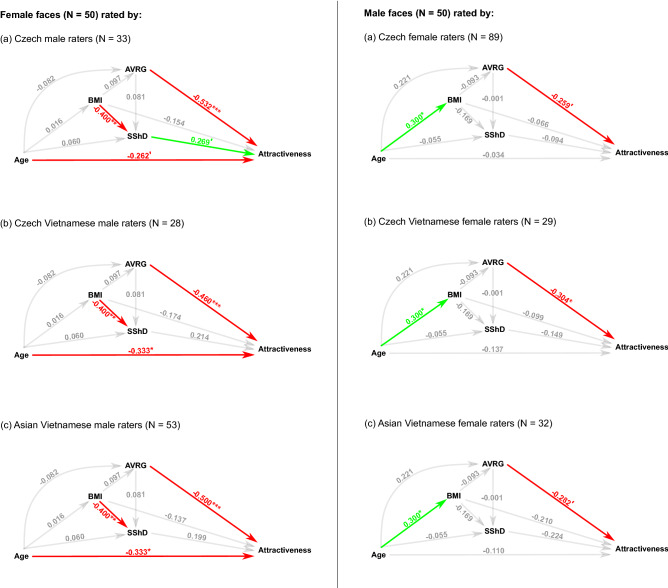
Visualization of path analyses of correlations between reported age, body mass index (BMI), sexual shape dimorphism (SShD), measured averageness (AVRG), and attractiveness. Arrows denote causal directions. The number next to a significant path describes the estimate of regression coefficient of the model with standardized variables. Green color of an arrow denotes a positive coefficient, red color a negative one. Asterisks represent the level of significance (p < 0.05*, p < 0.01**, p < 0.001***) of partial regression coefficient being non-zero. Gray arrows denote absence of a significant relationship. Apostrophe (‘) denotes a nonsignificant trend (p < 0.1, p > 0.05). The higher the SShD value, the more female sex-typical is the facial shape. Lower (negative) values correspond to more masculine facial configurations. A positive association between perceived facial attractiveness and SShD in male faces therefore means that less sex-typical male faces are perceived as more attractive. In female faces, a positive association between SShD and perceived attractiveness indicates that women with more sex-typical (feminine) faces are perceived as more attractive. In both sexes, the negative association between averageness and attractiveness means that more average faces were perceived as more attractive.

We used the path analysis to trace interrelations between attractiveness, age, BMI, SShD, and averageness. In total, we performed six analyses. We separately analyzed the perception of male and female Czech faces by three groups of raters, namely Czech Europeans, the Czech Vietnamese, and the Asian Vietnamese. The results are summarized in Fig. 1.

Path analyses yielded a constant cross-group structure of a causal pattern of interdependencies for both male and female Czech faces. Attractive female faces were closer to the population average. BMI and age had a negative effect on perceived facial attractiveness. In general, the effect of sex-typicality (here represented by SShD) on attractiveness was not statistically significant. Only Czech European male raters tended to be sensitive to female SShD: they associated attractiveness with more feminine-shaped faces. In the case of male faces, averageness influenced the perception of attractiveness but for Czech and Asian Vietnamese female raters, this association was not statistically significant. Otherwise, we found no other differences between Czech European, Czech Vietnamese, and Asian Vietnamese rater groups.

## Discussion

As expected, Czech Vietnamese raters were more accurate than Asian Vietnamese raters at predicting the attractiveness of Czech faces (whereby attractiveness of Czech faces as assessed by Czech European raters was considered a standard). In general, however, the results regarding preferences for averageness and sex-typicality did not correspond to the initial theoretical expectations.

The reason why Czech Vietnamese raters were capable of estimating facial attractiveness of Czech faces more accurately (again, with the Czech European judgments taken as a standard) than Asian Vietnamese raters may be in part due to differences in the perceptual narrowing in early childhood. Infants discriminate between faces of their own and other populations equally well until the age of six months. Then this capacity decreases and by the ninth month of life, young children are losing this ability. In later life, people thus discriminate among faces belonging to other populations less well than among faces belonging to their own population^87–90^. All Czech Vietnamese raters in our sample were born in the Czech Republic or moved to the Czech Republic at a very young age. Most of them should thus have some very early experience with European faces, for instance, during visits to a Czech doctor, while the Vietnamese residing in Vietnam would not be likely to have such early experiences of European faces. The better accuracy in the assessment of attractiveness might be thus due to differences in the perceptual narrowing between the Vietnamese born in the Czech Republic and those who were born and grew up in Vietnam.

Path analysis did not reveal any substantial differences between the three compared rater groups of either sex. Czech men tend to prefer more sex-typical women (r = 0.27). Still, the association was not statistically significant (at alpha = 0.05), and the mixed effects model also did not reveal any nonrandom association between SShD and attractiveness. Female facial attractiveness was negatively associated with age. Age did not significantly affect perceived attractiveness of Czech male faces when rated by women from all three rater-groups. These results support previous findings^69,72^. BMI and attractiveness were mediated by facial sex-typicality in female faces. Higher BMI negatively affected the morphological femininity of Czech female faces.

The degree of sexual dimorphism varies across different human populations^91^. In this context, it may be relevant to note that European faces express a greater level of sexual dimorphism than faces of Asian origin^92^. The lower degree of sexual dimorphism in Vietnamese persons in comparison to more dimorphic Czech Europeans could be the reason why for the Asian Vietnamese sexual dimorphism (facial masculinity/femininity) may be a less efficient cue for assessing overall facial attractiveness. An additional reason why Vietnamese raters of Asian origin did not tend to utilize traits associated with sexual shape dimorphism as a cue of attractiveness may be that Asian people tend to attribute relatively greater significance to facial skin color and texture than Europeans do^93^. Czech Vietnamese raters followed the same pattern as their Asian peers, which seems to contradict the abovementioned assumption of importance of environmental influence. One may speculate that genetic and/or parental imprinting-like effects might play a greater role in setting the preferences for sexually dimorphic facial traits.

A possible weakness of this study may be the relatively small sample size of female raters belonging to the Vietnamese minority in the Czech Republic. The Czech Vietnamese community is not very open to outsiders and especially female members of this community willing to participate in this research were hard to find. In future research, this limitation could be reduced, for instance, by monetary reward for participation to attract more Czech Vietnamese raters, although a promise of reward may be a selection criterion that could bias the understanding of attractiveness perception among this community. Ideally, future research should be undertaken by a Czech Vietnamese researcher who might be able to secure a higher level of cooperation of the Vietnamese diaspora living in the Czech Republic.

On the whole, we found that facial averageness of female European faces (in contrast to only weak or none effect in males) plays a significant role in attractiveness assessment in all three rater groups that differ in their racial background, country of residence, or both. This is partly consistent with the "average is attractive" hypothesis^41,43^. In both sexes, the place of long-term residence and/or upbringing was a significant factor in their perception of facial attractiveness. Vietnamese respondents living in Czech Republic were significantly better at predicting attractiveness of Czech faces than Vietnamese living in Vietnam were.

## Supplementary Information

Supplementary Information.

## Data Availability

The dataset and R code is available at https://osf.io/9a5mt/.

## References

[1] Müllerová P (1998). Vietnamese DIASPORA in the Czech Republic. Arch. Orient..

[2] Kleisner K, Chvátalová V, Flegr J (2014). Perceived intelligence is associated with measured intelligence in men but not women. PLoS ONE.

[3] Třebický V, Havlíček J, Roberts SC, Little AC, Kleisner K (2013). Perceived aggressiveness predicts fighting performance in mixed-martial-arts fighters. Psychol. Sci..

[4] Linke L, Saribay SA, Kleisner K (2016). Perceived trustworthiness is associated with position in a corporate hierarchy. Pers. Individ. Dif..

[5] Little AC, Třebický V, Havlíček J, Roberts SC, Kleisner K (2015). Human perception of fighting ability: Facial cues predict winners and losers in mixed martial arts fights. Behav. Ecol..

[6] Todorov A, Olivola CY, Dotsch R, Mende-Siedlecki P (2015). Social attributions from faces: Determinants, consequences, accuracy, and functional significance. Psychology.

[7] Schmälzle R (2019). Visual cues that predict intuitive risk perception in the case of HIV. PLoS ONE.

[8] Asch SE (1946). Forming impressions of personality. J. Abnorm. Soc. Psychol..

[9] Bar M, Neta M, Linz H (2006). Very first impressions. Emotion.

[10] Willis J, Todorov A (2006). First impressions: Making up your mind after a 100-ms exposure to a face. Psychol. Sci..

[11] Bothwell RK, Brigham JC, Malpass RS (1989). Cross-racial identification. Personal. Soc. Psychol. Bull..

[12] Meissner CA, Brigham JC (2001). Thirty years of investigating the own-race bias in memory for faces: A meta-analytic review. Psychol. Public Policy Law.

[13] Sporer SL (2001). Recognizing faces of other ethnic groups: An integration of theories. Psychol. Public Policy Law.

[14] Hugenberg K, Young SG, Bernstein MJ, Sacco DF (2010). The categorization-individuation model: An integrative account of the other-race recognition deficit. Psychol. Rev..

[15] Anzures G (2013). Developmental origins of the other-race effect. Curr. Dir. Psychol. Sci..

[16] Suhrke J (2014). The other-race effect in 3-year-old German and Cameroonian children. Front. Psychol..

[17] Sangrigoli S, de Schonen S (2004). Effect of visual experience on face processing: A developmental study of inversion and non-native effects. Dev. Sci..

[18] Scott LS, Monesson A (2009). The origin of biases in face perception. Psychol. Sci..

[19] Ma F, Xu F, Luo X (2015). Children’s and Adults}’ {Judgments of Facial {Trustworthiness}: The {Relationship} to Facial {Attractiveness}. Percept. Mot. Skills.

[20] Tanaka JW, Kiefer M, Bukach CM (2004). A holistic account of the own-race effect in face recognition: Evidence from a cross-cultural study. Cognition.

[21] Webster Michael A, MacLeod Donald IA (2011). Visual adaptation and face perception. Philos. Trans. R. Soc. B.

[22] Bukach CM, Cottle J, Ubiwa J, Miller J (2012). Individuation experience predicts other-race effects in holistic processing for both Caucasian and Black participants. Cognition.

[23] Třebický V (2018). Cross-{cultural} evidence for apparent {racial} outgroup {advantage}: Congruence between perceived {facial} aggressiveness and fighting {success}. Sci. Rep..

[24] Hebl MR, Williams MJ, Sundermann JM, Kell HJ, Davies PG (2012). Selectively friending: Racial stereotypicality and social rejection. J. Exp. Soc. Psychol..

[25] Cassidy KD, Quinn KA, Humphreys GW (2011). The influence of ingroup/outgroup categorization on same- and other-race face processing: The moderating role of inter- versus intra-racial context. J. Exp. Soc. Psychol..

[26] Johnson KJ, Fredrickson BL (2005). We all look the same to Mepositive emotions eliminate the own-race bias in face recognition. Psychol. Sci..

[27] Bernstein MJ, Young SG, Hugenberg K (2007). The cross-category effect: Mere social categorization is sufficient to elicit an own-group bias in face recognition. Psychol. Sci..

[28] Hugenberg K, Miller J, Claypool HM (2007). Categorization and individuation in the cross-race recognition deficit: Toward a solution to an insidious problem. J. Exp. Soc. Psychol..

[29] Little AC, Jones BC, DeBruine LM (2011). Facial attractiveness: Evolutionary based research. Philos. Trans. R. Soc. B..

[30] Langlois JH (2000). Maxims or myths of beauty? A meta-analytic and theoretical review. Psychol. Bull..

[31] Penton-Voak IS, Jacobson A, Trivers R (2004). Populational differences in attractiveness judgements of male and female faces: Comparing British and Jamaican samples. Evol. Hum. Behav..

[32] Saxton TK, Little AC, DeBruine LM, Jones BC, Roberts SC (2009). Adolescents' preferences for sexual dimorphism are influenced by relative exposure to male and female faces. Pers. Individ. Dif..

[33] Badaruddoza A (2007). A paradox of human mate preferences and natural selection. J. Hum. Ecol..

[34] Coetzee V, Greeff JM, Stephen ID, Perrett DI (2014). Cross-cultural agreement in facial attractiveness preferences: The role of ethnicity and gender. PLoS ONE.

[35] Hulse FS (1967). Selection for skin color among the Japanese. Am. J. Phys. Anthropol..

[36] Kleisner K (2017). African and European perception of African female attractiveness. Evol. Hum. Behav..

[37] Kleisner K, Priplatova L, Frost P, Flegr J (2013). Trustworthy-looking face meets brown eyes. PLoS ONE.

[38] Zebrowitz LA, Montepare JM, Lee HK (1993). They don't all look alike: Individual impressions of other racial groups. J. Pers. Soc. Psychol..

[39] Laeng B, Mathisen R, Johnsen JA (2007). Why do blue-eyed men prefer women with the same eye color?. Behav. Ecol. Sociobiol..

[40] Gründl M, Knoll S, Eisenmann-Klein M, Prantl L (2012). The blue-eyes stereotype: Do eye color, pupil diameter, and scleral color affect attractiveness?. Aesthetic Plast. Surg..

[41] Langlois JH, Roggman LA (1990). Attractive faces are only average. Psychol. Sci..

[42] Rhodes G, Tremewan T (1996). Averageness, exaggeration, and facial attractiveness. Psychol. Sci..

[43] Rhodes G (2006). The evolutionary psychology of facial beauty. Annu. Rev. Psychol..

[44] Thornhill R, Gangestad SW (1999). Facial attractiveness. Trends Cogn. Sci..

[45] Rhodes G (2001). Attractiveness of facial averageness and symmetry in non-western cultures: In search of biologically based standards of beauty. Perception.

[46] Langlois JH, Roggman LA, Musselman L (1994). What is average and what is not average about attractive faces?. Psychol. Sci..

[47] Baudouin JY, Tiberghien G (2004). Symmetry, averageness, and feature size in the facial attractiveness of women. Acta Psychol..

[48] Perrett DI, May KA, Yoshikawa S (1994). Facial shape and judgements of female attractiveness. Nature.

[49] Alley TR, Cunningham MR (1991). Averaged faces are attractive, but very attractive faces are not average. Psychol. Sci..

[50] Pittenger JB (1991). On the difficulty of averaging faces: Comments on Langlois and Roggman. Psychol. Sci..

[51] Komori M, Kawamura S, Ishihara S (2009). Averageness or symmetry: Which is more important for facial attractiveness?. Acta Psychol..

[52] Rhodes G, Sumich A, Byatt G (1999). Are average facial configurations attractive only because of their symmetry?. Psychol. Sci..

[53] Scott LS, Tanaka JW, Sheinberg DL, Curran T (2008). The role of category learning in the acquisition and retention of perceptual expertise: A behavioral and neurophysiological study. Brain Res..

[54] Komori M, Kawamura S, Ishihara S (2009). Effect of averageness and sexual dimorphism on the judgment of facial attractiveness. Vis. Res..

[55] Jones D, Hill K (1993). Criteria of facial attractiveness in five populations. Hum. Nat..

[56] Little AC, Connely J, Feinberg DR, Jones BC, Roberts SC (2011). Human preference for masculinity differs according to context in faces, bodies, voices, and smell. Behav. Ecol..

[57] Van den Berghe PL, Frost P (1986). Skin color preference, sexual dimorphism and sexual selection: A case of gene culture co-evolution?*. Ethn. Racial Stud..

[58] Fink B, Neave N, Seydel H (2007). Male facial appearance signals physical strength to women. Am. J. Hum. Biol..

[59] Scheib Joanna E, Gangestad Steven W, Randy T (1999). Facial attractiveness, symmetry and cues of good genes. Proc. R. Soc. Lond. Ser. B..

[60] Perrett DI (1998). Effects of sexual dimorphism on facial attractiveness. Nature.

[61] Penton-Voak IS (1999). Menstrual cycle alters face preference [7]. Nature.

[62] Rhodes G, Hickford C, Jeffery L (2000). Sex-typicality and attractiveness: Are supermale and superfemale faces super-attractive?. Br. J. Psychol..

[63] Kościński K (2007). Facial attractiveness: General patterns of facial preferences. Anthropol. Rev..

[64] Johnston VS, Hagel R, Franklin M, Fink B, Grammer K (2001). Male facial attractiveness: Evidence for hormone-mediated adaptive design. Evol. Hum. Behav..

[65] Scott IM (2014). Human preferences for sexually dimorphic faces may be evolutionarily novel. Proc. Natl. Acad. Sci..

[66] Brooks R (2011). National income inequality predicts women's preferences for masculinized faces better than health does. Proc. R. Soc. B.

[67] DeBruine LM, Jones BC, Little AC, Crawford JR, Welling LLM (2011). Further evidence for regional variation in women's masculinity preferences. Proc. R. Soc. Lond. B..

[68] Dunson DB, Colombo B, Baird DD (2002). Changes with age in the level and duration of fertility in the menstrual cycle. Hum. Reprod..

[69] Hassan MAM, Killick SR (2003). Effect of male age on fertility: Evidence for the decline in male fertility with increasing age. Fertil. Steril..

[70] Buss DM (1989). Sex differences in human mate preferences: Evolutionary hypotheses tested in 37 cultures. Behav. Brain Sci..

[71] Maestripieri D, Klimczuk ACE, Traficonte DM, Wilson MC (2014). A greater decline in female facial attractiveness during middle age reflects women's loss of reproductive value. Front. Psychol..

[72] McLellan B, McKelvie SJ (1993). Effects of age and gender on perceived facial attractiveness. Can. J. Behav. Sci. Can. Sci. Comport..

[73] Bovet J, Barkat-Defradas M, Durand V, Faurie C, Raymond M (2018). Women's attractiveness is linked to expected age at menopause. J. Evol. Biol..

[74] Coetzee V, Perrett DI, Stephen ID (2009). Facial adiposity: A cue to health?. Perception.

[75] Coetzee V, Chen J, Perrett DI, Stephen ID (2010). Deciphering faces: Quantifiable visual cues to weight. Perception.

[76] Schneider TM, Hecht H, Carbon CC (2012). Judging body weight from faces: The height-weight illusion. Perception.

[77] Grillot RL, Simmons ZL, Lukaszewski AW, Roney JR (2014). Hormonal and morphological predictors of women's body attractiveness. Evol. Hum. Behav..

[78] Hume DK, Montgomerie R (2001). Facial attractiveness signals different aspects of "quality" in women and men. Evol. Hum. Behav..

[79] Tovée MJ, Swami V, Furnham A, Mangalparsad R (2006). Changing perceptions of attractiveness as observers are exposed to a different culture. Evol. Hum. Behav..

[80] Třebický V, Fialová J, Kleisner K, Havlíček J (2016). Focal LENGTH AFFECTS DEPICTED SHAPE AND PERCEPTION OF FACIAL IMAGES. PLoS ONE.

[81] Kuznetsova A, Brockhoff PB, Christensen RHB (2017). lmerTest package: tests in linear mixed effects models. J. Stat. Softw..

[82] Hothorn T, Bretz F, Westfall P (2008). Simultaneous inference in general parametric models. Biometrical J..

[83] Rosseel Y (2012). Lavaan: An R package for structural equation modeling. J. Stat. Softw..

[84] Adams, D. C., Collyer, M. L. & Kaliontzopoulou, A. Geomorph: Software for geometric morphometric analyses. R package version 3.1.0. (2019).

[85] Mitteroecker P, Windhager S, Müller GB, Schaefer K (2015). The morphometrics of 'masculinity' in human faces. PLoS ONE.

[86] Valenzano DR, Mennucci A, Tartarelli G, Cellerino A (2006). Shape analysis of female facial attractiveness. Vis. Res..

[87] De Haan M, Pascalis O, Johnson MH (2002). Specialization of neural mechanisms underlying face recognition in human infants. J. Cogn. Neurosci..

[88] Kelly DJ (2009). Development of the other-race effect during infancy: Evidence toward universality?. J. Exp. Child Psychol..

[89] Krasotkina A, Götz A, Höhle B, Schwarzer G (2018). Perceptual narrowing in speech and face recognition: Evidence for intra-individual cross-domain relations. Front. Psychol..

[90] Kelly DJ (2007). Cross-race preferences for same-race. Infancy.

[91] Kleisner K (2020). How and why patterns of sexual dimorphism in human faces vary across the world.. Infancy.

[92] Hopper WJ, Finklea KM, Winkielman P, Huber DE (2014). Measuring sexual dimorphism with a race-gender face space. J. Exp. Psychol. Hum. Percept. Perform..

[93] Tan KW, Tiddeman B, Stephen ID (2018). Skin texture and colour predict perceived health in Asian faces. Evol. Hum. Behav..

