# Supplementary material for: Environmental convergence in facial preferences: a cross-group comparison of Asian Vietnamese, Czech Vietnamese, and Czechs
Source: Sci Rep. 2021 Jan 12;11:550. doi: 10.1038/s41598-020-79623-1 (PMC7804147; doi:10.1038/s41598-020-79623-1)
Supplement: Supplementary file 1 — Supplementary Information. [file 41598_2020_79623_MOESM1_ESM.docx]

**Supplementary Information:**

**Environmental convergence in facial preferences: A cross-group comparison of Asian Vietnamese, Czech Vietnamese, and Czechs**

Ondřej Pavlovič^1^, Vojtěch Fiala^1^, Karel Kleisner^1^*

^1^ Department of Philosophy and History of Science, Faculty of Science, Charles University, Vinicna 7, Prague 128 44, Czech Republic

*corresponding author: karel.kleisner@natur.cuni.cz

**Table S1** Pearson’s correlations between attractiveness ratings and anthropometric traits for photographs of men

| MEN | CZ_attr | CZVN_attr | VN_attr | SShD | AVRG | Age | BMI |
| --- | --- | --- | --- | --- | --- | --- | --- |
| Mean±  SD | 2.778± 0.866 | 2.521± 0.728 | 3.208± 0.613 | -0.019± 0.016 | 0.054± 0.012 | 24.040± 3.917 | 22.377± 2.273 |
| CZ_attr |  | **0.912** | **0.838** | -0.076 | -0.263 | -0.094 | -0.052 |
| CZVN_attr | 0.000 |  | **0.913** | -0.114 | -0.327 | -0.210 | -0.104 |
| VN_attr | 0.000 | 0.000 |  | -0.172 | -0.296 | -0.204 | -0.194 |
| SShD | 0.697 | 0.620 | 0.376 |  | -0.007 | -0.105 | -0.186 |
| AVRG | 0.195 | 0.107 | 0.129 | 0.960 |  | 0.193 | -0.026 |
| Age | 0.639 | 0.340 | 0.340 | 0.620 | 0.340 |  | 0.300 |
| BMI | 0.795 | 0.620 | 0.340 | 0.344 | 0.898 | 0.129 |  |

Notes: CZ_attr – attractiveness rated by Czech Europeans; CZVN_attr – attractiveness rated by the Czech Vietnamese, VN_attr – attractiveness rated by the Asian Vietnamese; SShD – sexual shape dimorphism; AVRG – distance from the average; BMI – Body Mass Index; Pearson’s correlation coefficients are shown above the diagonal axis; p-values below the diagonal axis has been adjusted for repeated tests by Benjamini-Hochberg correction; significant correlations are set in bold

**Table S2** Pearson’s correlations between attractiveness ratings and anthropometric traits for photographs of women

| WOMEN | CZ_attr | CZVN_attr | VN_attr | SShD | AVRG | Age | BMI |
| --- | --- | --- | --- | --- | --- | --- | --- |
| Mean± SD | 2.681± 0.858 | 2.566± 0.696 | 3.261± 0.518 | 0.019± 0.018 | 0.054± 0.013 | 23.640± 4.332 | 22.158± 2.901 |
| CZ_attr |  | **0.928** | **0.871** | 0.297 | **-0.515** | -0.209 | -0.314 |
| CZVN_attr | 0.000 |  | **0.898** | 0.249 | **-0.441** | -0.288 | -0.307 |
| VN_attr | 0.000 | 0.000 |  | 0.218 | **-0.478** | -0.286 | -0.268 |
| SShD | 0.076 | 0.123 | 0.179 |  | 0.038 | 0.047 | **-0.391** |
| AVRG | 0.001 | 0.005 | 0.002 | 0.833 |  | -0.081 | 0.095 |
| Age | 0.191 | 0.078 | 0.078 | 0.823 | 0.672 |  | 0.016 |
| BMI | 0.070 | 0.070 | 0.097 | 0.015 | 0.630 | 0.915 |  |

Notes: CZ_attr – attractiveness rated by Czech Europeans; CZVN_attr – attractiveness rated by the Czech Vietnamese, VN_attr – attractiveness rated by the Asian Vietnamese; SShD – sexual shape dimorphism; AVRG – distance from the average; BMI – Body Mass Index; Pearson’s correlation coefficients are shown above the diagonal axis; p-values below the diagonal axis has been adjusted for repeated tests by Benjamini-Hochberg correction; significant correlations are set in bold
